# Long noncoding RNA expression profile reveals lncRNAs signature associated with extracellular matrix degradation in kashin-beck disease

**DOI:** 10.1038/s41598-017-17875-0

**Published:** 2017-12-14

**Authors:** Cuiyan Wu, Huan Liu, Feng’e Zhang, Wanzhen Shao, Lei Yang, Yujie Ning, Sen Wang, Guanghui Zhao, Byeong Jae Lee, Mikko Lammi, Xiong Guo

**Affiliations:** 1 School of Public Health, Health Science Center of Xi’an Jiaotong University; Key Laboratory of Trace Elements and Endemic Diseases, National Health and Family Planning Commission of the People’s Republic of China, Xi’an, 710061 P.R. China; 2 Department of Knee Joint, Xi’an Hong Hui Hospital, Xi’an, 710054 P.R. China; 3 Institute of Molecular Biology and Genetics, School of Biological Sciences, Seoul National University, Seoul, 151742 Korea; 4 Department of Integrative Medical Biology, Umeå University, Umeå, 90187 Sweden

## Abstract

Kashin-Beck disease (KBD) is a deformative, endemic osteochondropathy involving degeneration and necrosis of growth plates and articular cartilage. The pathogenesis of KBD is related to gene expression and regulation mechanisms, but long noncoding RNAs (lncRNAs) in KBD have not been investigated. In this study, we identified 316 up-regulated and 631 down-regulated lncRNAs (≥ 2-fold change) in KBD chondrocytes using microarray analysis, of which more than three-quarters were intergenic lncRNAs and antisense lncRNAs. We also identified 232 up-regulated and 427 down-regulated mRNAs (≥ 2-fold change). A lncRNA-mRNA correlation analysis combined 343 lncRNAs and 292 mRNAs to form 509 coding-noncoding gene co-expression networks (CNC networks). Eleven lncRNAs were predicted to have cis-regulated target genes, including NAV2 (neuron navigator 2), TOX (thymocyte selection-associated high mobility group box), LAMA4 (laminin, alpha 4), and DEPTOR (DEP domain containing mTOR-interacting protein). The differentially expressed mRNAs in KBD significantly contribute to biological events associated with the extracellular matrix. Meanwhile, 34 mRNAs and 55 co-expressed lncRNAs constituted a network that influences the extracellular matrix. In the network, FBLN1 and LAMA 4 were the core genes with the highest significance. These novel findings indicate that lncRNAs may play a role in extracellular matrix destruction in KBD.

## Introduction

Kashin-Beck disease (KBD) is a deformative, endemic osteochondropathy that involves degeneration and necrosis of growth plates and articular cartilage^1–3^. It results in growth retardation, secondary osteoarthritis and disability in the advanced stages. As of 2013, there were 0.64 million patients with KBD and 1.16 million persons at risk in 377 counties in China^1^. The etiology of this disease is associated with environmental factors^4,5^, and genetic factors are also known to be involved^6–8^. Three major environmental factors contributing to KBD have been proposed: 1) endemic selenium deficiency, 2) serious cereal contamination by mycotoxin-producing fungi, and 3) high humic acid levels in drinking water. Ninety-seven up-regulated and down-regulated genes were identified in KBD peripheral blood mononuclear cells and their functions were related to metabolism, apoptosis, the cytoskeleton, immunity, cell movement and the extracellular matrix^9^. A comparison of gene expression profiles between KBD and normal chondrocytes showed 79 differentially expressed genes, including up-regulated pro-apoptotic genes such as Box, Bax, and Bak, and down-regulated genes, such as Bcl-2 and Bcl-XL^10^. Furthermore, when the 79 genes were ran through the Environmental Genome Project and Comparative Toxicogenomics Database, 73 genes were found to be closely related to environmental factors (unpublished results). Hence, the researchers proposed a pathogenesis model for KBD, namely, “environmental risk factors - environmental response genes - cartilage damage”.

Noncoding RNAs have been found to be a small fraction of the total RNA population, and they function directly as structural, catalytic or regulatory RNAs. Noncoding RNAs can be classified into two major groups based on their length, namely, short noncoding RNAs and long noncoding RNAs (lncRNAs)^11^. Recently, much attention has focused on lncRNAs because increasing evidence indicates that lncRNAs affect gene transcription through a number of regulatory processes. LncRNAs employ several mechanisms for gene regulation. For example, they can directly bind to target genes or act as scaffolds for transcription factors and histone modifiers to activate or inhibit the expression of target genes^12,13^. In addition, lncRNAs can also serve as competing endogenous RNAs (ceRNAs) to modulate the concentration and biological function of mRNAs/miRNAs^14,15^.

Breakthroughs in recent years have revealed numerous examples of lncRNAs involvement in normal development and disease. Multiple studies have shown the function and mechanisms of lncRNAs and long noncoding intergenic RNAs (lincRNAs) in the pathogenesis of osteoarthritis (OA). For example, they are related to cartilage injury by promoting cartilage extracellular matrix degradation in OA^16^. Cytokine IL-1 stimulation induced changes in the profiles of lncRNAs PACER, CILinc01, and CILinc02^17^. A significant down-regulation of miR-101 and up-regulation of lncRNA HOTTIP regulated cartilage development and degradation in the processes of endochondral ossification and osteoarthritic progression^18^. The role of lncRNAs and their overall contributions to the pathogenesis of KBD, a special type of osteochondropathy, are still unknown.

In this study, we compared the expression profiles of lncRNAs between KBD and normal articular cartilage. Several lncRNAs with differential expression were validated using quantitative reverse transcription polymerase chain reaction (qRT-PCR). Further bioinformatics analysis was used to explore the potential function, lncRNA-mRNA correlation and potential targets of the differentially expressed lncRNAs.

## Results

### LncRNA expression profile

**Figure 1:**
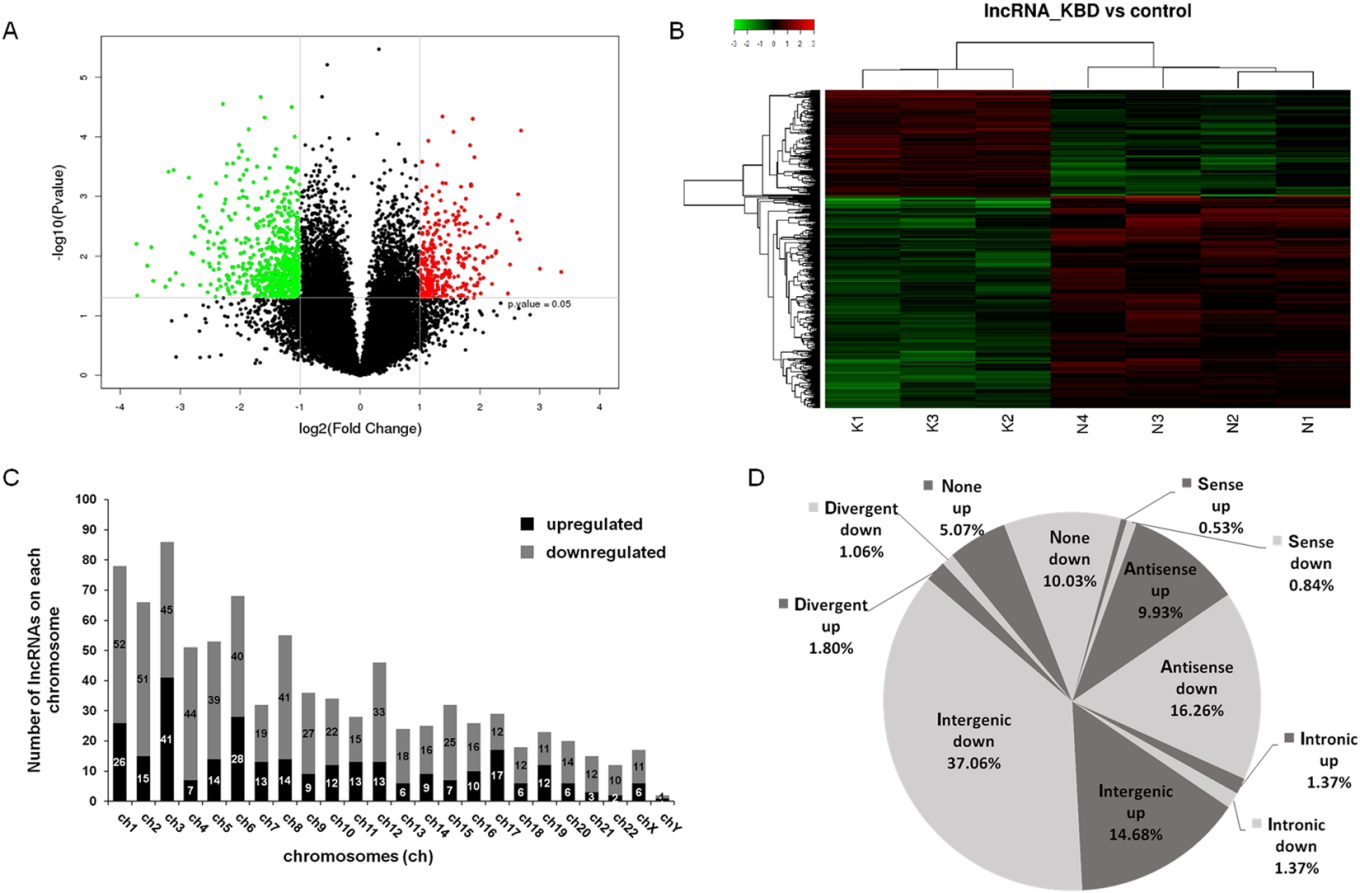
LncRNA profile based on microarray data. (**A**), Volcano plot of differentially expressed lncRNAs in KBD chondrocytes compared with normal controls. Red points represent significantly up-regulated and green points significantly down-regulated lncRNAs in KBD with a greater than 2.0-fold change. (**B**), Two-dimensional hierarchical clustering of distinguishable lncRNA expression profiles in KBD chondrocytes compared with normal controls. Red: higher expression levels; green: lower expression levels. Probes are in rows, and samples are in columns. (**C**), Distribution of differentially expressed lncRNAs in KBD, showing up-regulated (black) and down-regulated (gray) lncRNAs in each chromosome (ch). (**D**), Pie chart of differentially expressed lncRNAs identified in various subgroup categories.

The array analysis revealed that of 25,398 identified lncRNAs, 947 were differentially expressed in KBD chondrocytes compared with normal chondrocytes. Of these lncRNAs, 316 were up-regulated and 631 were down-regulated (Fig. 1A). Importantly, 30 lncRNAs were identified in KBD chondrocytes with fold change (FC) > 6.0 compared with normal chondrocytes (Table 1). The most up-regulated lncRNA was ENST00000531202.1 (FC = 24.347), and the most down-regulated lncRNA was TCONS_00015374 (FC = 13.283). Hierarchical clustering analysis indicated distinguishable lncRNA expression profile in KBD compared with that in normal controls (Fig. 1B). The differentially expressed lncRNAs in KBD chondrocytes were widely scattered among all chromosomes, although the distribution in the chromosomes was not equal (Fig. 1C). Chromosome 3 had the largest number of altered lncRNAs, with 41 up-regulated and 45 down-regulated lncRNAs, which accounted for 9.08% (86/947) of all the differentially expressed lncRNAs. Notably, there were 71 differentially expressed lncRNAs that could not be assigned to a corresponding chromosome.

**Table 1:** The differentially expressed lncRNAs in KBD chondrocytes.

lncRNA ID	Regulation	Fold change	P value	Corr P	Type
ENST00000531202.1	up	24.34733704	0.033989864	0.347510242	Antisense
TCONS_00028337	up	10.26061096	0.018442333	0.31251025	Intergenic
ENST00000437088.1	up	8.004592335	0.016324772	0.307282992	Intergenic
TCONS_00014631	up	6.436936142	7.86795E-05	0.183911455	Divergent
ENST00000502049.2	up	6.338411656	0.005233162	0.262757621	Antisense
RNA95045|RNS_127_194	up	6.226821012	0.000920774	0.199427973	none
ENST00000426475.1	up	6.154782189	0.004047084	0.248339279	Intergenic
TCONS_00015374	down	13.2834387	0.00621313	0.26647141	Intergenic
TCONS_00016355	down	13.16866872	0.045860274	0.373938714	Intergenic
ENST00000518941.1	down	11.71885041	0.014506312	0.303304503	Antisense
ENST00000511029.1	down	11.18167597	0.007102188	0.275661343	Intergenic
TCONS_00018333	down	10.91979168	0.025937269	0.331868145	Intergenic
XR_245446.2	down	9.482841504	0.03275995	0.346354485	none
XR_428239.1	down	9.162462986	0.000386058	0.19346474	none
ENST00000568735.1	down	9.025079384	0.024795142	0.330904046	Antisense
RNA146924|p0028_imsncRNA72	down	8.642969505	0.000361576	0.19346474	none
ENST00000422971.1	down	8.423308425	0.019243848	0.314710386	Antisense
ENST00000568302.1	down	7.773972489	0.030316937	0.340914143	Antisense
ENST00000439156.1	down	7.236713929	0.000484326	0.198602252	Intergenic
XR_430362.1	down	7.033541845	0.008872168	0.284800463	none
TCONS_00024285	down	6.902358307	0.009165309	0.285646994	Intergenic
ENST00000562678.1	down	6.763213584	0.004713153	0.255071797	Intergenic
ENST00000609012.1	down	6.444520671	0.001531114	0.210653141	Intergenic
XR_426818.1	down	6.366906717	0.000982015	0.199427973	none
HIT000061969	down	6.355408442	0.030592786	0.340925856	Intronic
ENST00000480669.1	down	6.330248493	0.003096341	0.234351521	Antisense
RNA96040|RNS_1122_80	down	6.295819281	0.000965452	0.199427973	none
TCONS_00018410	down	6.134346656	0.012098559	0.295808182	Intergenic
RNA95450|RNS_532_157	down	6.124923888	0.002149699	0.218654539	none
TCONS_00008985	down	6.103441806	0.003346962	0.238140075	Intergenic

### LncRNA classification and subgroup analysis

LncRNA classification and subgroup analysis were performed to explore the potential function of the differentially expressed lncRNAs. Differentially expressed lncRNAs were distributed among five subgroup types: sense, antisense, intronic, intergenic and divergent (Fig. 1D). The intergenic lncRNAs accounted for 51.7% of the differences (139/947 up-regulated and 351/947 down-regulated), while the antisense lncRNAs formed the second largest category (26.2%, 94/947 up-regulated and 154/947 down-regulated lncRNAs). Remarkably, approximately 15% of the lncRNAs could not be classified into any of the subgroups. We also identified 75 lncRNAs with enhancer-like functions among the differentially expressed lncRNAs (15 up-regulated and 60 down-regulated). In addition, 9 lincRNAs (3 up-regulated and 6 down-regulated) were identified.

### mRNA expression profile

**Figure 2:**
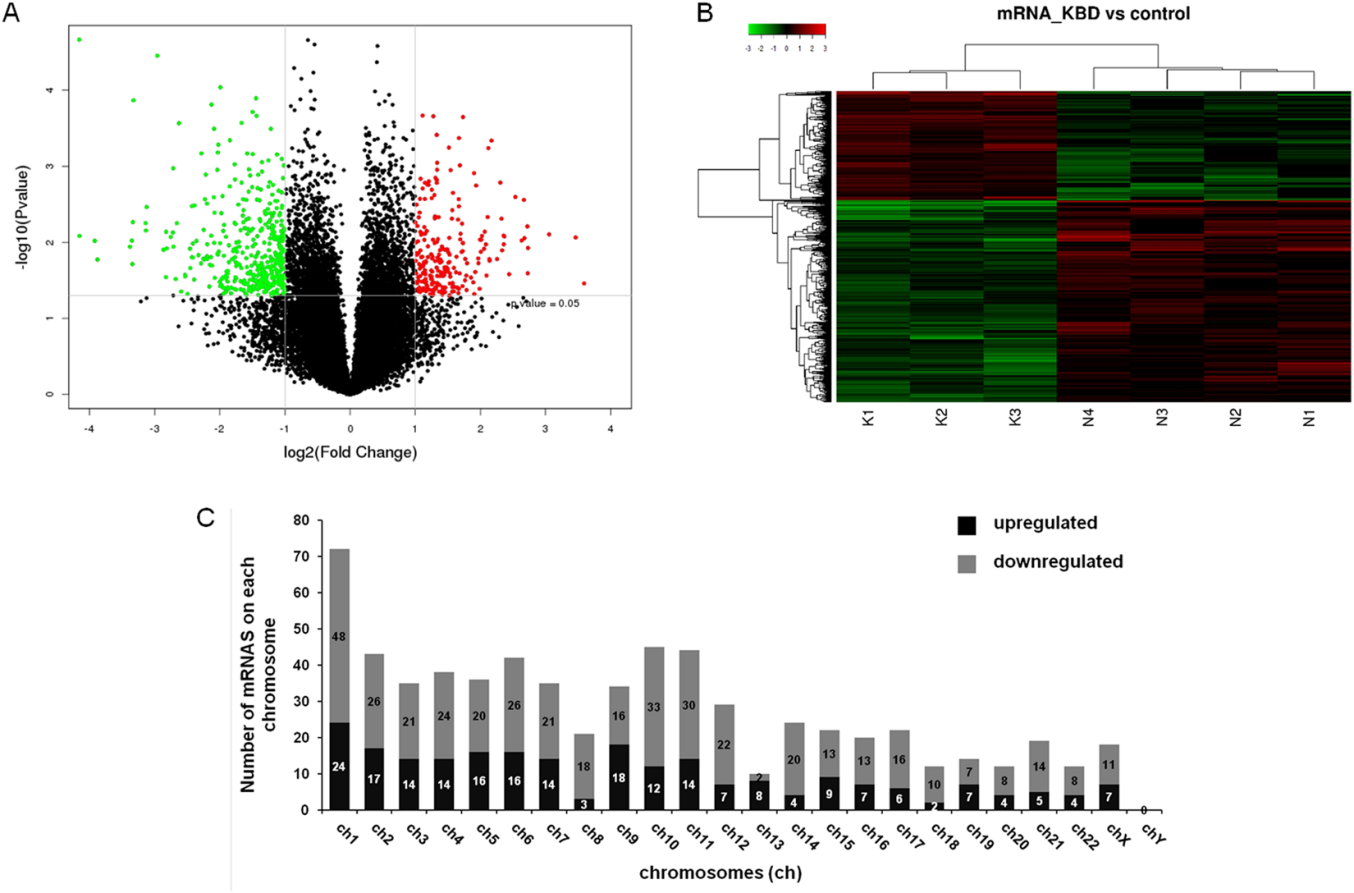
mRNAs profile of microarray data. (**A**), Volcano plot of differentially expressed mRNAs in KBD chondrocytes compared to normal controls. Red points represent significantly up-regulated and green points represent significantly down-regulated mRNAs in KBD with a greater than 2.0-fold change. (**B**), Two-dimensional hierarchical clustering of distinguishable mRNA expression profiles in KBD chondrocytes compared with normal controls. Red: higher expression levels; green: lower expression levels. Probes are in rows, and samples are in columns. (**C**), Distribution of differentially expressed mRNAs in KBD, showing up-regulated (black) and down-regulated (gray) mRNAs in each chromosome (ch).

The array analysis identified 26840 mRNAs, of which 232 were up-regulated and 427 down-regulated in KBD (Fig. 2A). The most up-regulated mRNA was TBX5 (FC = 43.140), and the most down-regulated mRNA was TBX20 (FC = 27.630). The array analysis identified 37 mRNAs that had FC > 6.0 in expression level between KBD and normal chondrocytes (Table 2). As shown in Tables 1 and 2, the Corr *P* value was slightly larger than the *P* value and more than 0.05. Considering the FDR requirement was so strict that it may miss a larger number of true alternatives, the Corr *P* was not used as the screening standard. Hierarchical clustering analysis indicated a distinguishable mRNA expression profile (Fig. 2B). Similar to the distribution pattern of lncRNAs, the differentially expressed mRNAs in KBD chondrocytes were widely but not equally scattered among all chromosomes, with the exception of the Y chromosome (Fig. 2C). Chromosome 1 had the largest number of differentially expressed mRNAs (24 up-regulated and 48 down-regulated), constituting up to 10.9% (72/659) of all the differentially expressed mRNAs.

**Table 2:** The differentially expressed mRNAs in KBD chondrocytes.

Genbank Accession	Gene-Symbol	Regulation	Fold change	P value	Corr P
NM_000192	TBX5	up	43.13953935	0.016108791	0.306966912
NM_001040214	NKAIN2	up	12.06780947	0.034717192	0.349182282
NM_017680	ASPN	up	11.02310687	0.008630902	0.281725889
NM_015393	PARM1	up	8.311360568	0.007868913	0.279587646
NM_002126	HLF	up	6.638579812	0.011867396	0.295505479
NM_207419	C1QTNF8	up	6.61998459	0.025534162	0.331045319
NM_033014	OGN	up	6.594546621	0.00618066	0.266170584
NM_033128	SCIN	up	6.391183329	0.008822067	0.284589239
NM_001193335	ASPN	up	6.355960311	0.002771679	0.228783786
NM_000395	CSF2RB	up	6.203647432	0.009461393	0.287742517
NM_001166220	TBX20	down	27.63028817	0.012585781	0.298295066
NM_001996	FBLN1	down	17.8630653	2.16659E-05	0.163944185
XR_172388	LOC100506737	down	17.81744952	0.008236039	0.280691821
NM_001080471	PEAR1	down	15.14003181	0.009565009	0.288215449
NM_000600	IL6	down	14.71113857	0.016853341	0.307955018
NM_014729	TOX	down	10.40933051	0.011452851	0.293289566
NM_003979	GPRC5A	down	10.20388058	0.009503494	0.287863363
None	None	down	10.14120352	0.019330159	0.314739824
None	None	down	10.09349664	0.005428071	0.262757621
NM_006486	FBLN1	down	10.02726503	0.000136058	0.183911455
NM_000596	IGFBP1	down	8.814923057	0.005607904	0.262757621
NM_019043	APBB1IP	down	8.776658192	0.007001017	0.275661343
NM_001164000	MECOM	down	8.717129188	0.003435252	0.239320319
NM_001996	FBLN1	down	7.779696861	3.52075E-05	0.167334805
NM_019043	APBB1IP	down	7.292097658	0.012475383	0.297831231
NM_033380	COL4A5	down	7.244044433	0.012429002	0.297831231
NM_198449	EMB	down	7.09166403	0.028707802	0.336971841
NM_198449	EMB	down	7.084398152	0.007280058	0.27706829
NM_153370	PI16	down	7.026795071	0.012025399	0.295808182
NM_000640	IL13RA2	down	6.746368693	0.008491194	0.281725889
NM_001297559	HTRA3	down	6.585869096	0.007446522	0.27706829
NM_001040058	SPP1	down	6.564959626	0.00106534	0.201692042
AF017464	None	down	6.553021835	0.011410641	0.293289566
XM_005249745	IL6	down	6.395766235	0.03034377	0.340914143
None	None	down	6.316393358	0.005617621	0.262757621
None	None	down	6.176727577	0.000272309	0.19346474
NM_003617	RGS5	down	6.034207422	0.044355767	0.370488491

### LncRNA-mRNA co-expression network

The co-expression correlation between lncRNAs and mRNAs in KBD was revealed by a CNC network, which indicated potential internal adjustment mechanisms (Figure S1 and Data S1). Among 1000 co-expressed pairs of related genes with the highest correlation coefficient, there were 343 lncRNAs and 292 mRNAs that constituted 509 network pairs. In the co-expression network, many lncRNAs were correlated with a single mRNA and vice versa. Among the network pairs, correlations were found between several top lncRNAs and mRNAs. For example, the top four down-regulated lncRNAs TCONS_00015374, TCONS_00016355, ENST00000511029.1, and TCONS_00018333 were correlated with the targets ENST00000511867 (no target gene symbol), ERICH2/DUSP4, LOC100506885 and OPALIN. The top second and third up-regulated lncRNAs were correlated with NXF2 and GRAMD2 (Table S2).

### LncRNA target prediction

To explore how lncRNAs may participate in gene regulation and the pathogenesis of KBD, cis- and trans- predictions were performed. Altogether, 11 lncRNAs were predicted to have cis-regulated target genes, of which down-regulated lncRNA ENST00000526642.1, ENST00000523683.1, and ENST00000588689.1 and up-regulated lncRNA ENST00000574086.1 were predicted to cis-regulate the genes NAV2 (neuron navigator 2, *P* = 1.24E-05), TOX (thymocyte selection-associated high mobility group box, *P* = 1.86E-05), LAMA4 (laminin, alpha 4, *P* = 1.41E-05) and DEPTOR (DEP domain containing mTOR-interacting protein, *P* = 1.90E-05), respectively.

### Differentially expressed mRNA and lncRNA profile related to extracellular matrix metabolism

GO analysis was performed first to more fully describe the roles of the differentially expressed mRNAs. The GO analysis included three categories: cellular components, molecular function and biological process. In this study, the most significantly enriched terms were proteinaceous extracellular matrix and extracellular matrix. In addition, the terms interstitial matrix, extracellular matrix component at the cellular components level, and extracellular matrix organization, extracellular structure organization at the biological process level were also significant. The input genes for the above terms were presented in Table S3 and included ADAMTS9, COL4A5, COL11A1, LRP4, LAMA4, NID2, LAMA1, MMP1, PRSS1, COL14A1, FBLN1, ITGA7, ASPN, MFAP2, FBLN2, COL8A2, SPP1, CTSK, ADAMTS9, PRSS1, SPP1, SLC3A1, DPP4, SLC7A8, CPB2, and PRSS1. The major pathways considered to involve differentially expressed mRNAs in KBD included extracellular matrix organization events, neuronal system events, and cell cycle events. Interestingly, the most significant pathway was extracellular matrix organization (http://www.reactome.org/cgi-bin/eventbrowser_st_id?ST_ID = REACT_118779), and within this category, another four pathways were related to extracellular matrix organization, such as laminin interactions, degradation of the extracellular matrix, collagen degradation and protein digestion and absorption.

The target gene-associated, differentially expressed lncRNAs were highly enriched for the most significant GO terms, including extracellular matrix (GO:0031012), laminin complex (GO:0043256), proteinaceous extracellular matrix (GO:0005578) and laminin-1 complex (GO:0005606). The most significantly enriched pathways were extracellular matrix proteoglycans (REACT_163906), non-integrin membrane-extracellular matrix interactions (REACT_163874) and laminin interactions (REACT_169262).

**Figure 3:**
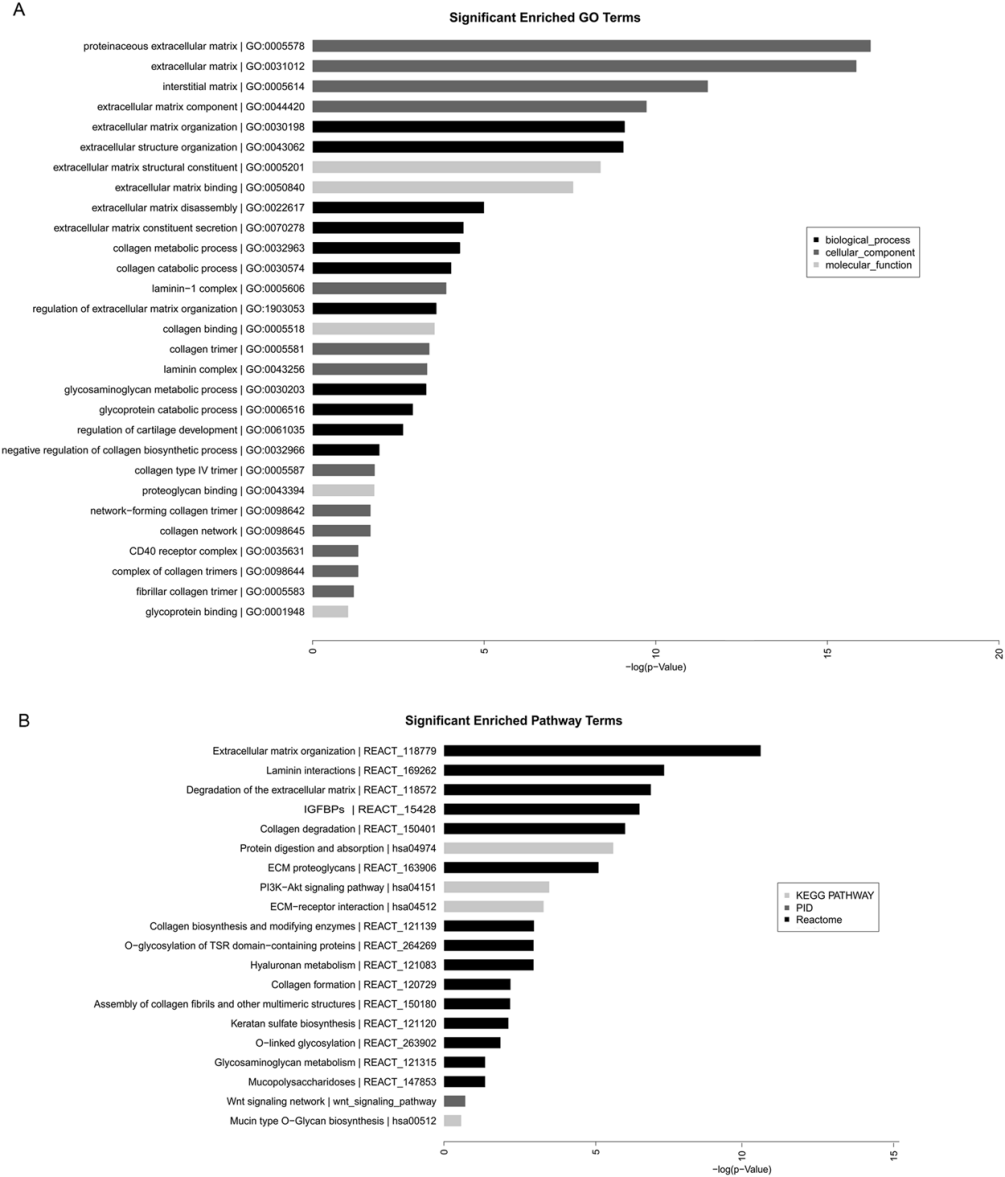
(**A**), Significantly enriched GO terms and (**B**), Significantly enriched pathway terms associated with extracellular matrix.

**Figure 4:**
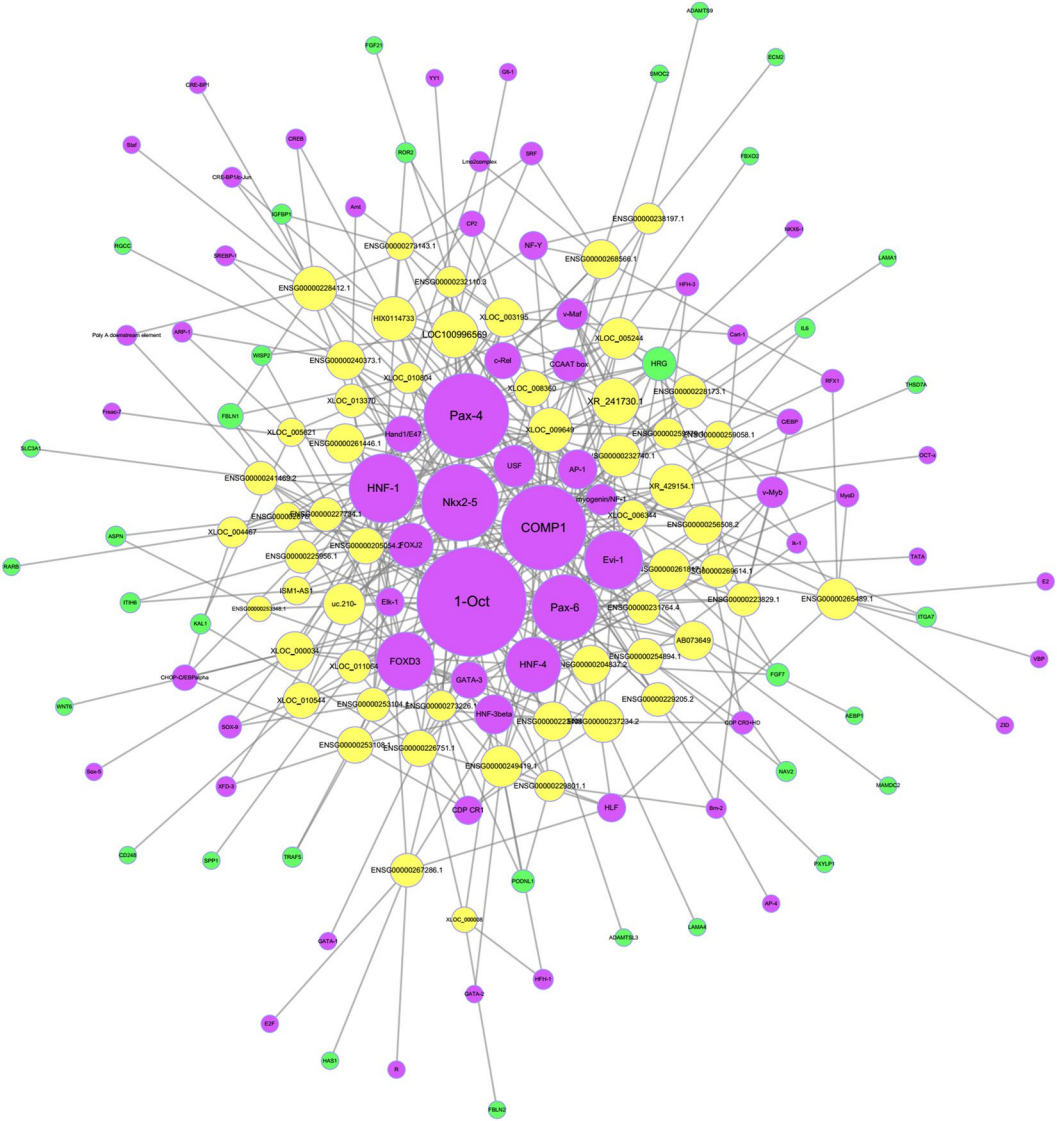
Network of differentially expressed lncRNAs, co-expressed genes and TFs associated with extracellular matrix. Yellow, green and purple spots represent lncRNAs, mRNAs and TFs, respectively.

Differentially expressed mRNAs associated with the extracellular matrix and their co-expressed lncRNAs were further analyzed. There were 34 differentially expressed mRNAs associated with extracellular matrix and 55 co-expressed lncRNAs, which involved in significantly enriched GO terms and significantly enriched pathway associated with extracellular matrix (Fig. 3). We also calculated and constructed a network of lncRNAs, co-expressed genes and transcription factors (TF) to identify common genes involved in lncRNA regulation (Fig. 4). In the list of genes and the network associated with the extracellular matrix, FBLN1 and LAMA4 were the core genes of highest significance. FBLN1 is a target gene of lncRNA ENSG00000227734.1 (name: RP11-49L2.1, Pearson coefficient = 0.995, *P* = 3.14E-06), and the related TFs included FOXD3, FOXJ2, HNF-1, Nkx2-5, Oct-1 and Pax-6. LAMA4 is a target gene of lncRNA ENSG00000237234.2 (name: RP1-142L7.5, Pearson coefficient = 0.991, *P* = 1.41E-05), and the related TFs included AP-1, CDP, CR1, CDP, CR3 + HD, COMP1, Evi-1, FOXD3, HNF-3, Nkx2-5 and Oct-1.

### Confirmation of differentially expressed lncRNAs using qRT-PCR

To validate the reliability of the lncRNA microarray data, we selected three up-regulated lncRNAs (RNA95045|RNS_127_194, ENST00000426475.1, and ENST00000437088.1) and three down-regulated lncRNAs (XR_245446.2, ENST00000568735.1, and ENST00000568302.1) that were abundantly expressed and exhibited significant changes (FCs  > 6.0) and used qRT-PCR to analyze differences in their expression. The qRT-PCR analysis results were mostly consistent with the microarray data (Figure S2).

## Discussion

Abnormal expression of lncRNAs has been observed to be involved in the pathogenesis and progression of many diseases by regulating gene expression profiles. Some studies have investigated the expression and function of lncRNAs associated with OA^16–20^. However, the pattern of lncRNAs expression and function in terms of the development and pathogenesis of KBD has not been investigated previously. Our study first screened the genome-wide expression pattern of lncRNAs and mRNAs in chondrocytes from KBD patients and normal subjects. Thereafter, we systematically analyzed the characteristic lncRNAs profile associated with KBD by comparing the differentially expressed lncRNAs and mRNAs between KBD chondrocytes and normal controls using bioinformatic methods.

The results showed that a high number of lncRNAs and mRNAs display abnormal expression in KBD chondrocytes. Altogether, 947 lncRNAs and 659 mRNAs were identified to be significantly differentially expressed in KBD. Overall, more lncRNAs and mRNAs were down-regulated than up-regulated. Several significantly differentially expressed lncRNAs were chosen for qRT-PCR validation. The expression of lncRNAs validated by qRT-PCR showed little disagreement compared with the microarray results. The discrepancies in RNA expression level based on microarray analysis and qRT-PCR are frequent and logical^21,22^. These discrepancies may be partially explained by differences in the two methods because they utilize vastly different normalization procedures and other different inherent pitfalls^23^.

We found that differentially expressed lncRNAs were not equally distributed across all chromosomes. Compared with other chromosomes, chromosomes 1, 2 and 3 had a higher percentage of differentially expressed lncRNAs and mRNAs. Although KBD is not a genetic disease, it has certain hereditary susceptibility^24^. Individuals whose parents and siblings suffer from KBD are at 3- to 4-fold higher risk of KBD than random non-related individuals^25^. Five short tandem repeat (STR) units on chromosome 2 have been shown to be correlated with the risk of KBD^26^. Therefore, chromosomes 1, 2 and 3 may be more likely to carry lncRNAs susceptible to KBD pathology. Interestingly, we also found that more lncRNAs and mRNAs were transcribed from chromosome X than from chromosome Y.

According to differences in transcriptional form, lncRNAs can be classified into subgroups of sense lncRNAs, antisense lncRNAs, intronic lncRNAs, intergenic lncRNAs, and divergent lncRNAs. Strikingly, we found a high proportion of intergenic lncRNAs and antisense lncRNAs, 51.74% and 26.19%, respectively, which together accounted for more than three-quarters of the differentially expressed lncRNAs identified. LincRNAs are transcribed from regions of at least 5 kb, from protein-coding genes. They can modulate the expression of target genes, and the target genes can be scattered across the genome^27^. Antisense lncRNAs are transcribed against and overlap with the protein-coding genes and regulate their protein-coding counterparts via multiple mechanisms. The many abnormally expressed intergenic lncRNAs and antisense lncRNAs in KBD indicate that lncRNAs may regulate protein-coding genes during KBD progression. In addition, lincRNA has high tissue specificity, even more specific than protein coding genes^28^. Based on this high specificity, lincRNA has become an excellent descriptor of different cell subsets for diagnostic purposes, including diseased cells. Therefore, in future studies, we may focus on the attribution of lncRNA to the auxiliary diagnosis of KBD.

LncRNAs can regulate the expression of their adjacent or overlapping genes through multiple mechanisms and are often transcribed together with their associated target genes. Thus, to a certain extent, the function of lncRNAs may be reflected by the function of their associated target genes. Based on the GO and pathway analyses of mRNA, lncRNAs and protein-coding genes, many dysregulated mRNAs in KBD chondrocytes were identified to contribute to degeneration of articular cartilage by regulating extracellular matrix organization, laminin interactions, degradation of the extracellular matrix, collagen degradation, and protein digestion and absorption. These genes include collagen type 4 alpha 5 (COL4A5), COL8A2, COL11A1, COL14A1, extracellular matrix protein 1 (ECM1), ECM2, matrix metallopeptidase 1 (MMP1), laminin subunit alpha 1 (LAMA1), LAMA4, fibulin 1 (FBLN1), FBLN2, osteoglycin (OGN), nidogen 2 (NID2), Wnt family member 6 (WNT6), asporin (ASPN), integrin subunit alpha 7 (ITGA7), hyaluronan synthase 1 (HAS1), and cathepsin K (CTSK).

Similar to degenerative OA, excessive degeneration of the cartilage extracellular matrix is a significant pathological feature of KBD^3,29,30^. Proteoglycan and collagens are the major macromolecules in cartilage extracellular matrix. Decreased proteoglycan content can be found in the deep zone of the cartilage of KBD patients, particularly in necrotic areas^31^. Aggrecan generated epitopes present in KBD cartilage and increased the CD44 level in cartilage and the sCD44 level in serum. Type II collagen expression is decreased and type I and III collagen expression increased in KBD cartilage^32^. Type II collagen telopeptides, potential markers of cartilage damage, are increased in serum^33^. Recent studies have demonstrated that lncRNAs are abnormally expressed in OA cartilage, and lncRNA-CIR was related to cartilage injury by promoting cartilage extracellular matrix degradation. Specific lncRNAs for cartilage extracellular matrix degradation in KBD will be verified *in vitro* in future studies, including lncRNA RP11-49L2.1 and lncRNA RP1-142L7.5.

KBD is related to environmental factor-gene interactions; for instance, T-2 toxin reduced the mRNA expression of aggrecan, collagen II and Bcl2 and increased the mRNA expression of p53, caspase-3, and Bax in chondrocytes^34–36^. Selenium deficiency up-regulated the mRNA expression of p53, caspase-3 and Bax and down-regulated Bcl2 mRNA expression in chondrocytes of selenium-deficient rats^36^. These results are consistent with the excessive chondrocyte apoptosis observed in KBD cartilage. However, there have not been any reports that the risk factors of KBD directly act on lncRNA in chondrocytes. To date, there is also no evidence showing a dynamic relationship between environmental factors and the expression of mRNAs or lncRNAs.

In conclusion, this study is the first to present the lncRNA expression profile of chondrocytes from KBD patients. The results suggested that abnormal lncRNAs are key regulators of gene expression and have important biological effects, especially in cartilage extracellular matrix degradation. The precise mechanism will be confirmed by further studies to contribute to the understanding of KBD pathogenesis and identify relevant biomarkers.

## Methods

### Subjects and sample size

Articular cartilage samples were obtained from KBD patients undergoing joint replacement surgery and normal donors who died in traffic accidents. Radiographs of the subjects’ right hand were taken, and the KBD patients were diagnosed as second degree or third degree based on the Diagnosing Criteria of Kashin-Beck Disease in China (WS/T 207–2010). The normal controls were from non-KBD-prevalent areas, and individuals with KBD, OA, rheumatoid arthritis or other bone and cartilage diseases were excluded, based on information provided by relatives. The sample size for the microarray analysis was five vs. five. The data of two KBD patients and one normal control were eliminated based on cluster analysis, and thus, the sample size for data analysis was three KBD (two females and one male, age range 55–70 years) vs. four normal controls (two females and two males, age range 50–66 years). Certain difficulties in obtaining cartilage tissue limited the sample size.

### Articular cartilage collection and chondrocyte culture

The KBD cartilage samples were collected after operation and the normal control group samples were collected from fresh cadaver knees within 10 hours of death. The cartilage samples in the KBD group and the normal control group were obtained from the same anatomical site. The cartilage tissues were cut into 3–5 mm^3^ slices and digested with trypsin and type II collagenase to isolate primary chondrocytes. After being washed in PBS, the cells were cultured in DMEM/F-12 (1:1) supplemented with 10% (v/v) fetal calf serum, 100 units/ml penicillin, and 100 μg/mL streptomycin at 37 °C in 5% CO_2_. The confluent cells were harvested at the first passage using 0.25% trypsin with 0.08% EDTA for experiments.

### RNA extraction

Total RNA was extracted from cultured chondrocytes derived from cartilage using TRIzol reagent (Invitrogen, USA) and purified with a mirVana miRNA Isolation Kit (Ambion, Austin, TX, USA) according to the manufacturer’s instructions. The purity and concentration of RNA were determined by assessing OD260/280 using a spectrophotometer (NanoDrop ND-1000). RNA integrity was determined with 1% formaldehyde denaturing gel electrophoresis.

### RNA labeling and hybridization

Total RNA was amplified and reverse transcribed into fluorescent cDNA using a CapitalBio cRNA Amplification and Labeling Kit (CapitalBio, Beijing, China) to produce high yields of Cy3- and Cy5-labeled cDNAs. The controls were labeled with Cy3-dCTP, and the KBD samples were labeled with Cy5-dCTP. After confirmation of the quality and quantity of the labeled products, they were used for microarray hybridization.

### Microarray analysis

LncRNA and mRNA expression profiling were performed using Agilent human lncRNA + mRNA array 4.0 platform (4 × 180 K), with each array containing approximately 41,000 lncRNA and 34,000 mRNA probes. LncRNA and mRNA target sequences were merged from multiple databases, such as GENCODE/ENSEMBL, Human LincRNA Catalog and many others. The microarray analysis was performed by CapitalBio Technology, Beijing, China.

### Microarray imaging and data analysis

The acquired microarray images were obtained using Agilent Feature Extraction (v10.7) software. Summarization, normalization and quality control of the original data were performed using GeneSpring software V13.0 (Agilent). The *P* value was calculated based on t-test and the corrected *P* value (Corr *P*) was calculated based on Benjamini-Hochberg controlled false discovery rate (FDR). Both lncRNAs and mRNAs were considered to significantly differ when the absolute FC value was more than 2.0 and t-test *P* value was equal to or less than 0.05. Furthermore, hierarchical clustering with average linkages were applied and tree visualization was performed using Java Treeview to present diacritical lncRNA and mRNA expression patterns among the samples.

### LncRNA-mRNA correlation analysis

A lncRNA-mRNA correlation analysis was accomplished to identify significantly co-expressed lncRNAs and mRNAs with the standard of a Pearson correlation >0.99 or <−0.99 and *P* value < 0.05 using the open source bioinformatics software Cytoscape. A coding-noncoding gene co-expression network (CNC network) was constructed based on correlation analysis between differentially expressed lncRNAs and mRNAs.

### Target prediction

Target prediction, including cis- and trans-predictions, was performed based on the results for co-expressed lncRNAs and mRNAs; mRNAs were considered cis-regulated target genes when the Pearson correlation coefficient was >0.99 or <−0.99 and the mRNA loci were within 10 kb of each other along a group of expressed protein-coding genes. Thus, “cis” refers to the regulatory mechanisms in the same locus (not necessarily same allele), which include antisense-mediated regulation by lncRNAs of protein-coding genes that are encoded in the same locus. The trans-prediction was conducted using the Standalone BLAT v. 35 × 1 fast sequence search command line tool (http://hgdownload.cse.ucsc.edu/admin/exe/) to compare the full sequence of lncRNA with the 3′UTR of its co-expressed mRNAs.

### Transcription factor (TF) prediction

TF prediction was performed based on the results of co-expression using the prediction tool Match-1.0 Public. It predicted a situation in which regions within the 2000 bp upstream and 500 bp downstream of each lncRNA could bind to TFs. For each lncRNA, the overlaps (and their significance) for the co-expressed mRNA set and the TF target genes were calculated.

### GO analysis and pathway analysis

GO analysis was derived from Gene Ontology (www. geneontology.org) that provided three structured networks of defined terms describing the attributes of genes and gene products. The analysis method was based on Fisher’s exact test and calculation of *P* values. The *P* value denotes the significance of GO Term enrichment in a differentially expressed mRNA list. Pathway analysis was performed for differentially expressed mRNAs based on database. For both GO and pathway analyses, *P* < 0.05 was considered statistically significant.

### qRT-PCR analyses

Isolated RNA was reverse-transcribed to cDNA using a Reverse Transcription Kit (Takara, Dalian, China). The qRT-PCR analyses were performed using a StepOnePlus RT-PCR Instrument with Power SYBR Green (Takara, Dalian China). The qRT-PCR conditions were as follows: 95 °C for 2 minutes, followed by 40 cycles of 95 °C for 15 seconds and 60 °C for 30 seconds. All experiments were performed and analyzed in triplicate. The primers used in this study were listed in Table S1. Then, lncRNA expression levels were normalized to GAPDH and calculated using the 2^−**ΔΔ**Ct^ method.

### Ethics Statement

This study was approved by the Human Ethics Committee of Xi’an Jiaotong University, and performed according to the principles of the Declaration of Helsinki as revised in 1983. Written informed consent was obtained from all subjects or the relatives of donors.

## Electronic supplementary material

Supplementary files

figure S1

Dataset 1
