# Supplementary material for: Long noncoding RNA expression profile reveals lncRNAs signature associated with extracellular matrix degradation in kashin-beck disease
Source: Sci Rep. 2017 Dec 14;7:17553. doi: 10.1038/s41598-017-17875-0 (PMC5730583; doi:10.1038/s41598-017-17875-0)
Supplement: Supplementary file 1 — Supplementary files [file 41598_2017_17875_MOESM1_ESM.pdf]

Long Noncoding RNA Expression Profile Reveals lncRNAs Signature Associated with Extracellular Matrix Degradation in Kashin-Beck Disease

Cuiyan Wu[1], Huan Liu[1], Feng'e Zhang[1], Wanzhen Shao[1], Lei Yang[1], Yujie Ning[1], Sen Wang[1], Guanghui Zhao[2], Byeong Jae Lee[3], Mikko Lammi[4]*, Xiong Guo[1]*

[1.] School of Public Health, Health Science Center of Xi'an Jiaotong University; Key Laboratory of Trace Elements and Endemic Diseases, National Health and Family Planning Commission of the People's Republic of China, Xi'an, 710061, P. R. China.

[2.] Department of Knee Joint, Xi'an Hong Hui Hospital, Xi'an, 710054, P. R. China.

[3.] Institute of Molecular Biology and Genetics, School of Biological Sciences, Seoul National University, Seoul, 151742, Korea.

[4.] Department of Integrative Medical Biology, Umeå University, Umeå, 90187, Sweden.

*Correspondence and requests for materials should be addressed to Xiong Guo (email: guox@mail.xjtu.edu.cn) and Mikko Lammi (email: mikko.lammi@umu.se)

Supplementary Figures

(Note: The figure is too large to be clearly displayed here and it shows as a single PDF file.)

Figure S1. The co-expressed correlation between lncRNAs and mRNAs in KBD was revealed by CNC network. The yellow spots represent lncRNAs and the green spots represent mRNAs. The size of the circles represents the relative importance of the genes in the network. Red lines show a positive interaction between transcripts and blue lines show a negative interaction.

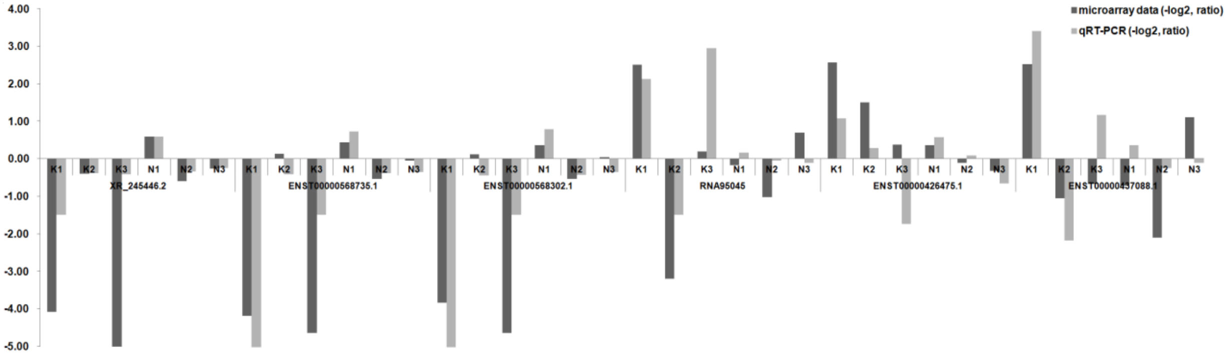

Figure S2. Validation of lncRNAs microarray data by qRT-PCR. The relative expression level of each lncRNA was normalized to GAPDH.

Supplementary Tables

Table S1. Primers used in qRT-PCR.

LncRNA ID	Forward	Reverse
XR_245446.2	TCCTTTTGTGGTAAATGCTTATTCT	AGGGAGGGAACAGCCTACAT
ENST00000568735.1	AATTGTACCTTTCCCGGCCA	TAGAATATGCCTGGCGGCTG
ENST00000568302.1	TTCCCGGCCAAGAAACCTC	GTGACTTCTTTAGCTGGCGG
RNA95045|RNS_127_194	ATGGGTCTGCCCTTGAGTT	TGTGAACGGAACCCACTGC
ENST00000426475.1	AATGCAGCCATTTGTCACCC	GCAGCAAAAGCTGAAGTGCTAA
ENST00000437088.1	GGTAGAAGCTGCTGTCCCTTAG	GCTGAGAAATCTCATGCCTGC

Table S2. Coding-non-coding networks pairs.

LncRNA Source	Target	Target Gene Symbol	Correlation	P-value
ENST00000531202.1	NM_022053	NXF2	0.996639665	1.25E-06
TCONS_00015374	ENST00000511867	---	0.997394017	6.65E-07
TCONS_00016355	*NM_001290030*	ERICH2	-0.990230209	1.80E-05
TCONS_00016355	*NM_001394*	DUSP4	-0.991685676	1.21E-05
TCONS_00016355	ENST00000511867	---	0.992357415	9.77E-06
ENST00000511029.1	*ENST00000607810*	LOC100506885	-0.991083246	1.44E-05
TCONS_00018333	NM_001040103	OPALIN	0.990295126	1.77E-05
ENST00000568735.1	ENST00000416507	---	0.997673597	5.01E-07
ENST00000422971.1	XR_172388	LOC100506737	0.993139299	7.46E-06
ENST00000437088.1	*NM_001012642*	GRAMD2	0.990117784	1.85E-05

The genes lncRNAs written in *italics* have up-regulated expression in KBD, while those written in normal font show the down-regulated gene expression.

Table S3. The GO terms and input genes associated with extracellular matrix

GO accession	Term	*P* value	Input gene symbols
GO:0005578	proteinaceous extracellular matrix*	8.66e-08	*ADAMTS9, PODNL1, COL11A1,* NID2, LAMA1, *COL8A2,* FBLN1, AMTN, FBLN2, *KAL1,* EGFLAM, NAV2, *OGN,* ECM1, *SMOC2,* WNT6, MMP1, LAMA4, CD248, *ADAMTSL3,* MFAP2, MAMDC2, COL4A5, COL14A1, *ECM2,* SERPINF1, *OMD,* WISP2, *ASPN*
GO:0031012	extracellular matrix*	1.31e-07	*ADAMTS9, PODNL1, COL11A1,* NID2, LAMA1, *COL8A2,* FBLN1, AMTN, AEBP1, FBLN2, *KAL1,* EGFLAM, NAV2, OGN, RARRES2, ECM1, *SMOC2,* WNT6, MMP1, LAMA4, CD248, *ADAMTSL3,* MFAP2, *PCSK6,* MAMDC2, COL4A5, COL14A1, *ECM2,* SERPINF1, *OMD,* WISP2, *ASPN*
GO:0005614	interstitial matrix*	9.95e-06	EGFLAM, COL14A1, *ECM2,* NAV2, *SMOC2,* MAMDC2
GO:0044420	extracellular matrix component*	5.94e-05	COL4A5, *COL11A1,* EGFLAM, LAMA4, NID2, *COL8A2,* LAMA1, COL14A1, FBLN1, AMTN, MFAP2, *SMOC2,* SERPINF1
GO:0043062	extracellular structure organization#	1.16e-04	*SMOC2, COL11A1,* NID2, LAMA1, FBLN1, ITGA7, FBLN2, PRSS1, EGFLAM, *COL8A2,* MMP1, LAMA4, *RGCC,* MFAP2, *LRP4,* COL4A5, DPP4, COL14A1, *ECM2,* HAS1, SPP1, *CPB2, MPZL3,* CTSK
GO:0030198	extracellular matrix organization#	1.11e-04	The same as upper row

The genes written in *italics* have up-regulated expression in KBD, while those written in normal font show the down-regulated gene expression. * cellular component; # biological process.
