# Supplementary material for: Long noncoding RNA expression profile reveals lncRNAs signature associated with extracellular matrix degradation in kashin-beck disease
Source: Sci Rep. 2017 Dec 14;7:17553. doi: 10.1038/s41598-017-17875-0 (PMC5730583; doi:10.1038/s41598-017-17875-0)
Supplement: Supplementary file 2 — figure S1 [file 41598_2017_17875_MOESM2_ESM.pdf]

Long Noncoding RNA Expression Profile Reveals lncRNAs Signature Associated with Extracellular Matrix Degradation in Kashin-Beck Disease

Cuiyan Wu[1], Huan Liu[1], Feng'e Zhang[1], Wanzhen Shao[1], Lei Yang[1], Yujie Ning[1], Sen Wang[1], Guanghui Zhao[2], Byeong Jae Lee[3], Mikko Lammi[4]*, Xiong Guo[1]*

[1.] School of Public Health, Health Science Center of Xi'an Jiaotong University; Key Laboratory of Trace Elements and Endemic Diseases, National Health and Family Planning Commission of the People's Republic of China, Xi'an, 710061, P. R. China.

[2.] Department of Knee Joint, Xi'an Hong Hui Hospital, Xi'an, 710054, P. R. China.

[3.] Institute of Molecular Biology and Genetics, School of Biological Sciences, Seoul National University, Seoul, 151742, Korea.

[4.] Department of Integrative Medical Biology, Umeå University, Umeå, 90187, Sweden.

*Correspondence and requests for materials should be addressed to XiongGuo (email: guox@mail.xjtu.edu.cn) and MikkoLammi (email: mikko.lammi@umu.se)

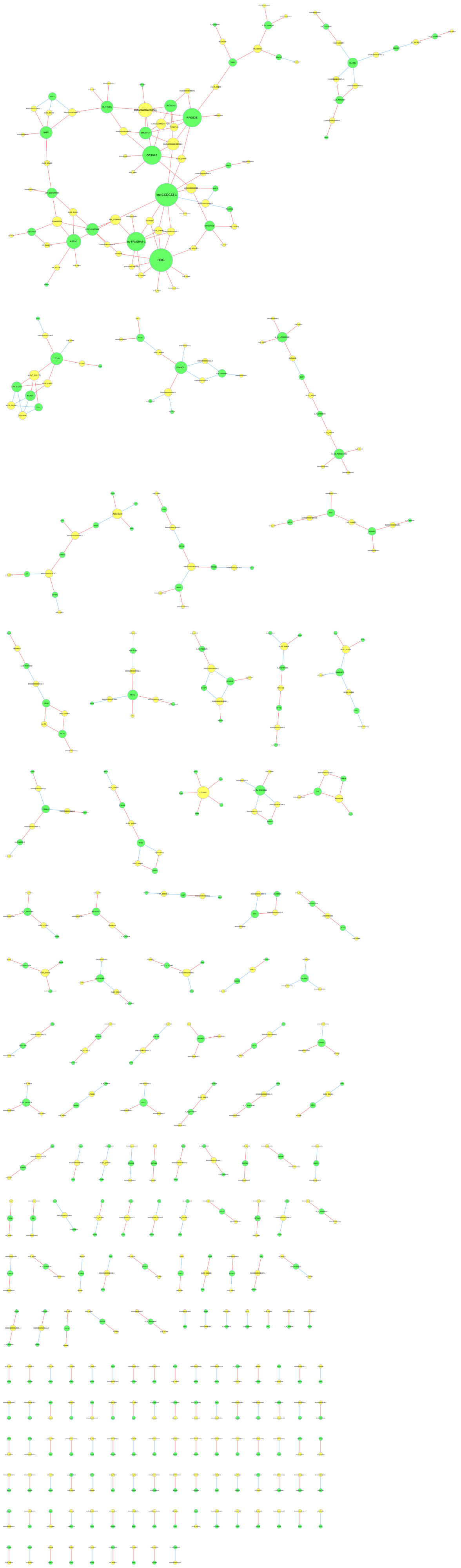

lnc-CCDC33-1
HRG
PAGE2B
OR10A2
lnc-FAM19A5-1
HLA-DQB1
LINC01102
ENSG00000224095.1
